# Nicotine dependence and smoking topography in tobacco-naïve users of disposable e-cigarettes: A prospective single-center, single-arm interventional study

**DOI:** 10.18332/tid/236207

**Published:** 2026-11-06

**Authors:** Elena Strohmeyer, Malina Kleine, Nadja Mallock-Ohnesorg, Nestor Burgmann, Franzen Klaas, Emily Firschke, Marcus Gertzen, Gabriele Koller, Annika Mahnke, Susanna Muckenhuber-Sternbauer, Anna Rahofer, Gabriel Roselli de Oliveira Pinto Kise, Katharina Sternberg, Andreas Luch, Elke Pieper, Tobias Rüther, Ariel Turcios, Andrea Rabenstein

**Affiliations:** 1 Department of Psychiatry and Psychotherapy, LMU University Hospital, Munich, Germany; 2 German Federal Institute for Risk Assessment (BfR), Department of Chemical and Product Safety, Berlin, Germany; 3 Institute of Legal Medicine, Goethe-University Frankfurt, Frankfurt/Main, Germany; 4 Medical Clinic III, Campus Lübeck, University Hospital Schleswig-Holstein, Lübeck, Germany; 5 Airway Research Centre North, Member of the German Centre for Lung Research (DZL), Großhansdorf, Germany; 6 Psychiatry and Psychotherapy, Faculty of Medicine, University of Augsburg, Augsburg, Germany

**Keywords:** nicotine addiction, FTND, disposable e-cigarettes, PSECDI, tobacco naïve

## Abstract

**INTRODUCTION:**

Currently, e-cigarettes constitute a major product category that enjoys substantial popularity, particularly with adolescents. Literature, however, requires studies conducted with tobacco-naïve youth and hence this single-center, one-arm study, aimed at characterizing this population, their consumption patterns, and the addictive potential of e-cigarettes.

**METHODS:**

Between December 2023 and August 2024, seventeen tobacco-naïve, regular e-cigarette users (>6 sessions/week) participated in this prospective single-arm interventional study at LMU University Hospital. Participants used a disposable e-cigarette ad libitum for 30 minutes during a 60 minute testing period. The results were compared with those from a previous adjacent study of occasional smokers using both a disposable e-cigarette and a combustible cigarette. We investigated nicotine dependence using two questionnaires: the Fagerström test for nicotine dependence (FTND) adapted for e-cigarettes and the Penn State Electronic Cigarette Dependence Index (PSECDI). Consumption behavior was characterized through puff topography using a smoking puff analyzer (SPA-M) and by repeated measurement of plasma nicotine concentrations.

**RESULTS:**

The mean FTND score was 3.29 (SD=2.47), corresponding to the questionnaire-defined category of moderate dependence (score 3–4). The mean PSECDI score was 7.71 (SD=3.96), corresponding to the questionnaire-defined category of low dependence (score 4–8). This suggests that regular e-cigarette use may lead to measurable nicotine dependence in a tobacco-naïve population. Ad libitum e-cigarette use was characterized by a larger time interval (seconds) between puffs (p=0.001; 95% CI: 24.97–81.29) compared to disposable e-cigarette consumption within a 5-minute time period. Nevertheless, nicotine concentrations consistently increased until the 30 minutes mark, reaching a final mean of 16.74 ng/mL, which is comparable to tobacco cigarettes.

**CONCLUSIONS:**

These results provide evidence that frequent e-cigarette use may induce nicotine dependence in previously tobacco-naïve individuals, highlighting the need for further characterization of this population and larger prospective studies to generate more robust evidence.

## INTRODUCTION

Since the introduction of e-cigarettes to the market, their popularity has been steadily increasing. This trend is reflected in the recent German Study on Tobacco Use (*Deutsche Befragung zum Rauchverhalten*, DEBRA study), which has tracked e-cigarette consumption in Germany since 2016^1^. While the use of conventional cigarettes among young adults (aged 18–24 years) steadily declined from 2023 to 2025 (from 37.6% to 32.3%), their consumption of e-cigarettes increased (from 3.5% to 4.1%)^1^.

A similar increase was observed in Great Britain, where the number of adult e-cigarette users rose from 0.8 to 5.6 million in 12 years (between 2012 and 2024)^2^. Moreover, consumption drastically rose for youth aged 11–17 years, with an increase of 7% (from 11% to 18%) of ever use since 2021. Monitoring the future (MTF), an English national survey monitoring the e-cigarette consumption in secondary school students since 2017 also recorded an increase and reported nicotine vaping as one of the most used drugs in all grades^3^. E-cigarette marketing directed at youth is deemed to be one possible reason for the mentioned increase, which makes e-cigarettes exceedingly popular within this age group^4^. An example of targeted marketing campaigns to young nicotine-naïve individuals can be found on product websites, where vapes are described with product statements like ‘perfect for beginners’^5^. Action on Smoking and Health (ASH) highlights that vapes are indeed used as an entry-level product. In 2022 ASH showed that e-cigarettes had overtaken traditional tobacco cigarettes as the most common entry product among those aged 11–17 years^6^. Various sources thus suggest that e-cigarettes are widely used as an introductory product, particularly by young and tobacco-naïve consumers, highlighting the need for studies in this population.

However, e-cigarettes are not only marketed as entry-level products but also as transition products for smokers intending to replace tobacco cigarettes. Some manufactures imply selling a form of nicotine replacement product, stating that due to its simple operation, their vapes have helped many smokers quit tobacco cigarettes and are ensuring that former smokers have a reliable alternative to tobacco cigarettes^5^. Moreover, many consumers perceive e-cigarettes as a product that can help to improve health^7^. In a questionnaire conducted by ASH, 50% of respondents agreed with the statement: ‘Vaping is a medicine that I use in order to address my smoking addiction’^2^. However, it is crucial to distinguish between nicotine replacement therapy (NRT) products and e-cigarettes. While e-cigarettes and their aerosols are currently considered less harmful than conventional cigarettes^8,9^, classifying them as harmless/NRT products would be problematic, given the scarcity of long-term studies. Besides, there is no official recognition as such in Germany. In numerous studies, the chemical composition of e-liquids and the chemicals formed in e-cigarette aerosol was examined highlighting potential health risks^10,11^. These widely used e-liquids contain humectants such as propylene glycol (PG) and vegetable glycerin (VG), nicotine, flavoring agents, and additives^12^.

The present study aims to evaluate the possible gateway potential and public health impact of these products by examining nicotine delivery, dependence, and craving. Literature requires studies conducted with tobacco-naïve youth; a closer look at this population seems highly relevant and a necessary basis for further research. While some researchers have focused on initiation into e-cigarette use^12^, this study aims at describing the population further. The main objective was therefore to draw conclusions about addictive behaviors and risks for regular, tobacco-naïve users. It was expected that participants would show measurable nicotine addiction, determined by the Penn State Electronic Cigarette Dependence Index (PSECD)^13^ and the Fagerström test of nicotine dependence (FTND)^14^ which was adapted for e-cigarette use.

Further, in a previous study, we showed that disposable e-cigarettes can deliver nicotine in a bolus cigarette-like delivery profile with sharp plasma nicotine increases^15^. However, ad libitum studies report varying nicotine curve shapes with an overall slower increase over time^16,17^. This discrepancy highlights the relevance of ad libitum studies to gain generalizable data and conclusive evidence to which delivery profile is closer to real-world consumption. Hence, we hypothesized that the smoking topography of dependent e-cigarette users, consuming ad libitum for 30 minutes, would differ from smoking topography of intermittent tobacco smokers. Detailed hypotheses are provided in the Supplementary file.

## METHODS

### Ethics and study products

This single-center study was performed in accordance with the principles of the Declaration of Helsinki and approved by the Ethics Committee at the Medical Faculty of Ludwig-Maximilians-University (LMU) Munich (No. 22-1069). Written informed consent was obtained from participants prior to the study. Additionally, travel and subject insurance were covered by ECCLESIA Mildenberger HOSPITAL GmbH. All participant contact took place between December 2023 and August 2024 at LMU University Hospital, Department of Psychiatry and Psychotherapy, Munich, Germany. Prior to data collection, participants were given comprehensive information about the study in a screening interview. The screening included study procedure, examination of inclusion and exclusion criteria, information about possible risks and data protection. After consent and inclusion, sociodemographic data were collected. On the following test day, participants then completed one session with the product they chose from preselection.

While we recognize the diversity of products, the different liquids, and further influencing factors such as the user himself^18^, we chose Elfbar, as it is one of the most popular e-cigarette brands worldwide due to its growing influence on the market, especially among adolescent and nicotine-naïve beginners^19-22^.

Nicotine levels refer to the product information. Products were purchased online and in stores in Germany from January to May 2023 and participants chose flavoring freely to imitate real-life consumption.

### Participants

A total of 29 young adults (aged 18–29 years) were recruited via personal contacts, e-mail circulars, social media, leaflets, and posters. All participants were regular e-cigarette users and defined themselves as ongoing consumers for ≥3 months, with ≥6 vaping sessions weekly. They further had to be tobacco-naïve participants (never smokers) – using a limit of 100 cigarettes within lifetime and no concurrent or dual use of other nicotine or tobacco products, specifically shisha. Nicotine abstinence was required at least 12 h before the examination. Therefore, participants with a baseline plasma nicotine level above 10 ng/mL were excluded from the study. For a comprehensive list of all inclusion and exclusion criteria, please refer to the Supplementary file. A convenience sample approach was used because the study required participants from a very specific target population who met stringent inclusion criteria.

### Study design

All study visits were performed indoors, and consumption took place ad libitum within a 30-minute time frame. Therefore, participants could use the product without any restrictions or puffing regime. At predefined times, blood samples were taken, hemodynamic parameters were measured, and questionnaires were completed. The overall testing period was 60 minutes. Study design is visualized in Figure 1.

**Figure 1:**
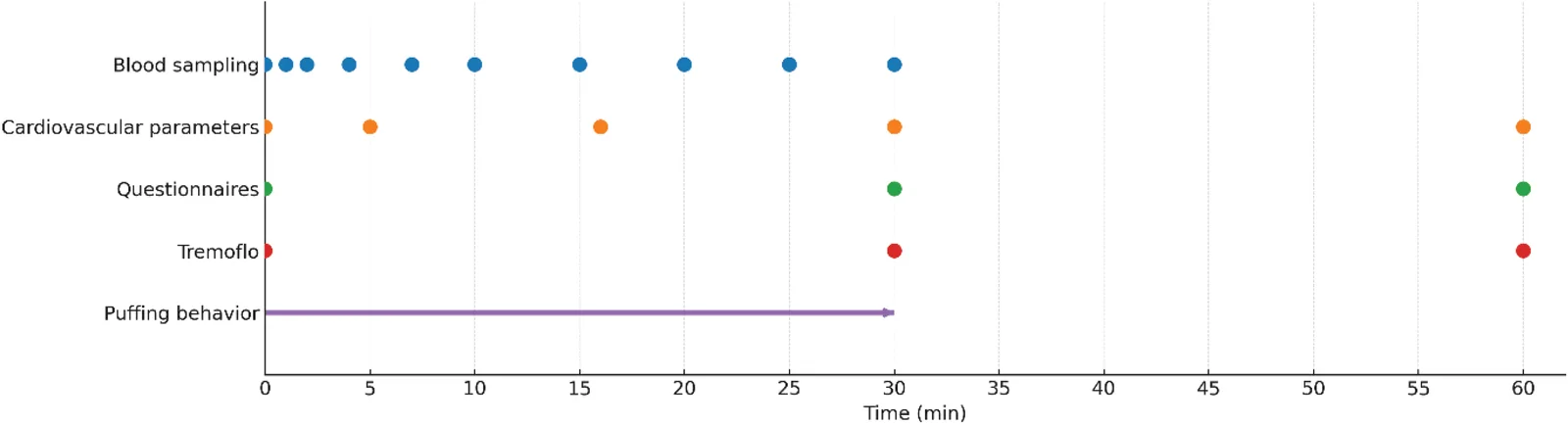
Study design overview with time points and measurements of the prospective single-center, one-arm pre-post interventional study conducted at the Department of Psychiatry and Psychotherapy, LMU University Hospital, 2024 (N=17)

### Questionnaires

Prior to the study procedure, addiction measures were queried using two questionnaires. The Fagerström test for nicotine dependence (FTND), a widely used and validated instrument that evolved from the original Fagerström Tolerance Questionnaire^14^, adapted for e-cigarettes, assessed the intensity of physical dependence on nicotine with six items. It included items such as: ‘How soon after waking up do you vape for the first time?’. The complete questionnaire is to be found in the Supplementary file. Additionally, the Penn State Electronic Cigarette Dependence Index (PSECDI) was used, an electronic cigarette adaptation of the Penn State Nicotine Dependence Index (PSNDI), which is a validated ten-item instrument designed to assess measurable nicotine dependence across all types of nicotine products^13^.

For simplicity reasons, craving was queried on a 7-point Likert-type scale after each blood sample. Further, the e-cigarette adapted 10-item version of The Questionnaire on Smoking Urges (QSU-U), was completed before and after consumption and used as a validated questionnaire. Factor analysis confirmed a two-factor structure: desire and intention to smoke as well as anticipation of relief from negative affect, and showed high internal consistency^23^. After the consumption had ended, participants had to rate their motivation to immediately consume the tested product again on a scale 1–7.

To record side effects, participants completed a questionnaire with a scale of: ‘none’, ‘mild’, ‘moderate’ and ‘severe’. This included subjectively perceived effects in mouth/throat, salivation, cold hands/feet, sweating, difficult inspiration, dizziness, headache, palpation, nausea and feeling of vomiting.

### Analysis of nicotine

Blood samples were collected using an indwelling venous catheter at ten given times: at baseline (0 minutes) and at 1, 2, 4, 7, 10, 15, 20, 25 and 30 minutes after starting consumption. The catheter was placed on the non-dominant hand whenever possible while the participant used the other hand for holding the e-cigarette. Blood samples were stored at 4–8°C until they were centrifuged and an internal standard solution was added to the plasma. Storage until shipment on dry-ice was at 80°C. The further analysis took place at the German Federal Institute for Risk Assessment (BfR) in Berlin, Germany, with a validated analytical method using liquid chromatography with tandem mass spectrometry (LC-MS/MS) after protein precipitation^24^. The baseline was set to 0 mg/mL for all participants.

### Smoking topography

The Smoking Puff Analyzer – Mobile (SPA-M, Sodim, ORT, France) was used to examine the vaping topography^25,26^. This portable device was interposed between e-cigarette and the mouth throughout the 30 minutes of consumption. It recorded the flow rate, pressure drop profile, and atmospheric pressure of smoking. Number of puffs, puff volume, puff duration, average flow per puff, peak flow per puff, the interval between puffs, and the smoking duration were analyzed using the software SodAfc41 – Version 4.02.0 (Sodim, ORT, France).

### Hemodynamic parameters and lung function

Hemodynamic parameters were assessed at baseline (0 minutes) and at 5, 16, 30 and 60 minutes after starting the consumption. The Mobil-O-Graph™ (software version HMS CS 4.2, I.E.M. GmbH) was utilized for measurement and calculation of heart rate (HR), central blood pressure (cBP), peripheral blood pressure (pBP), and parameters of arterial stiffness [total vascular resistance (TVR), pulse wave velocity (PWV), and the augmentation index adjusted for heart rate 75 bpm (AIX@75)]. Results are to be found in an adjacent study.

Two devices were used to assess lung function. At the initial screening, a spirometer was utilized to measure classical lung function parameters such as forced expiratory volume in 1 s (FEV_1_) and forced vital capacity (FVC). Additionally, lung function (specifically airway reactivity and resistance), was measured using Airwave Oscillometry (AO) with the Tremoflo device. These measurement methods differ primarily in their procedure. Airwave Oscillometry does not require forced inspiration or forced expiration, and measurements with the Tremoflo device were performed before the onset of vaping, immediately after the vaping period, and after a 30-minute intervention-free phase. Results are to be found in an adjacent study and the data are not yet fully analyzed.

### Statistical analysis

In an adjacent earlier study^15^, participants were recruited as conventional cigarette smokers (n=17) and smoked both a single tobacco cigarette and, on a consecutive test day, a tobacco-flavored e-cigarette. E-cigarette consumption was limited to 5 minutes. In the following analysis, the raw data are used for comparison purposes and referred to as ‘tobacco cigarette’ and ‘5-min e-cigarette’.

The software SPSS27 was used for statistical analyses. Means, standard deviations (SDs), and ranges were calculated for participant characteristics. Frequential statistics were used to assess differences between groups, with a significance threshold of 5%. Puffing behavior was compared between studies using Student’s independent-samples t-test, whereas the Wilcoxon rank-sum test was used to analyze craving because the data were not normally distributed. Nicotine delivery over time was analyzed using repeated-measures ANOVA with Greenhouse–Geisser correction to account for violations of the sphericity assumption.

## RESULTS

### Participant characteristics

While 29 participants were screened, only 25 participants attended testing at visit 2 and a further 8 participants were excluded due to missing blood samples or nicotine at baseline being >10 ng/mL. The drop out due to technical malfunctions can be considered at random. A full overview including detailed reasons for drop out can be found in the Supplementary file. Hence, 8 male and 9 female (n=17) subjects participated in this study, all within early adulthood with mean age 21.82 years (SD=2.65). The mean time since starting e-cigarette consumption at testing date was 33.59 months (SD=23.00).

**Table 1:** Tested disposable e-cigarette products with individual flavor choices used in a prospective single-center, one-arm pre-post interventional study conducted at the Department of Psychiatry and Psychotherapy, LMU University Hospital, 2024 (N=17)

*Product type*	*Flavor*	*Nicotine content (mg/mL)*
Disposable e-cigarette	Blueberry Raspberry	20
Disposable e-cigarette	Watermelon	20
Disposable e-cigarette	Pink Lemonade	20
Disposable e-cigarette	Tobacco	20
Disposable e-cigarette	Lemon Tart	20
Disposable e-cigarette	Menthol	20

Current at-home product use, assessed by the number of daily sessions, ranged 3–30, with the most common response being 10–15 sessions per day. Eleven participants reported Elfbar as their usual e-cigarette of choice, and 15 participants reported usually using products with a nicotine concentration of 20 mg/mL. The remaining two participants reported using products with nicotine concentrations of 10 mg/mL and 4 mg/mL, respectively. Flavor choice on testing day can be found in the Supplementary file.

### Nicotine dependence and subjective effects

Addiction measures were queried on two scales and revealed similar results (Figure 2). The mean FTND score was 3.29 (SD=2.47), corresponding to the questionnaire-defined category of moderate dependence (score 3–4). The mean PSECDI score was 7.71 (SD=3.96), corresponding to the questionnaire-defined category of low dependence (score 4–8). There was a positive correlation between those two scales (r=0.61, p=0.009). Participants frequently had difficulties recalling consumption frequency, duration, as well as number of puffs per session.

**Figure 2:**
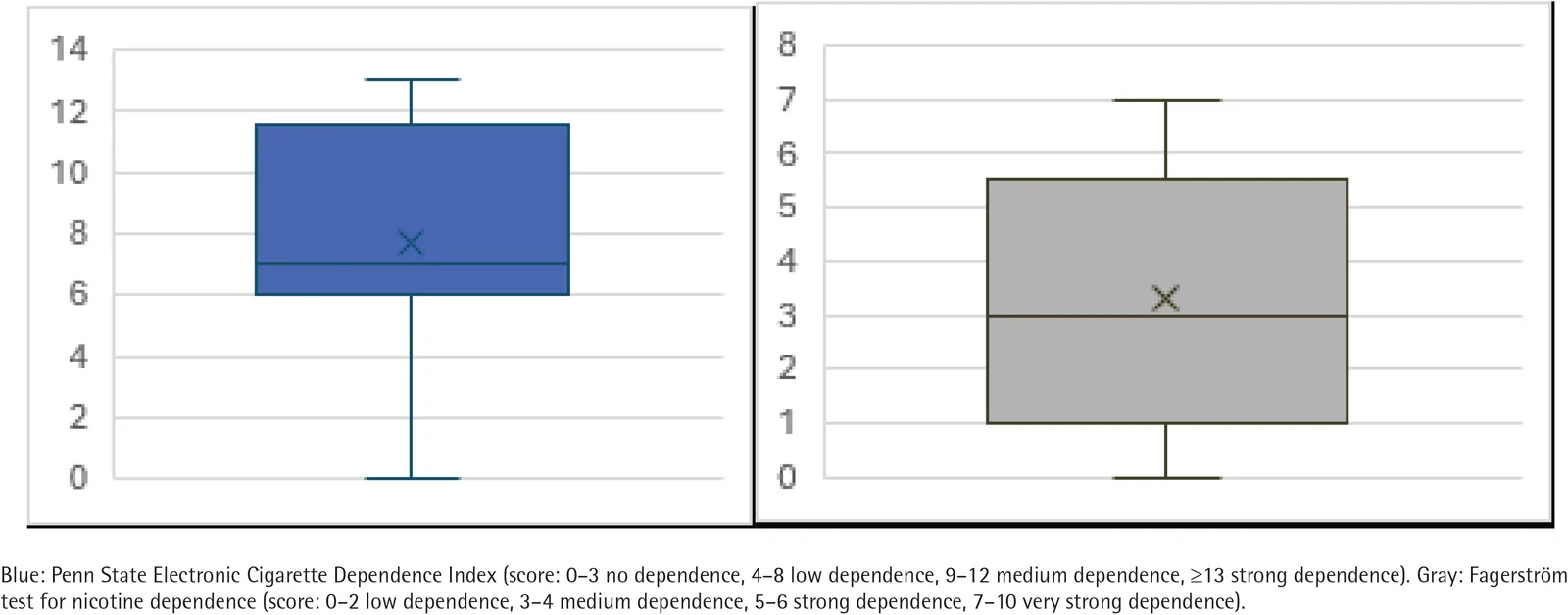
Boxplot of the extent of nicotine dependence queried in the prospective single-center, one-arm pre-post interventional study conducted at the Department of Psychiatry and Psychotherapy, LMU University Hospital, 2024 (N=17)

QSU results for craving were internally consistent (α=0.91 pre- and α=0.88 post-consumption) and the Wilcoxon test for non-normally distributed items revealed a significant reduction in craving (p<0.001; 95% CI: 0.53–1.55). This finding was supported by the single item craving query, which revealed a positive association between nicotine delivery and reduction in craving (p<0.001; 95% CI: -0.11 – -0.06).

### Nicotine delivery and plasma nicotine concentrations

Due to the ad libitum regimen, nicotine increased significantly in blood for the duration of testing (Greenhouse-Geisser p<0.001) and hence Cmax was not determined. Figure 3 shows the individual nicotine delivery and concentration profile of all participants, during 30 minutes of ad libitum consumption.

**Figure 3:**
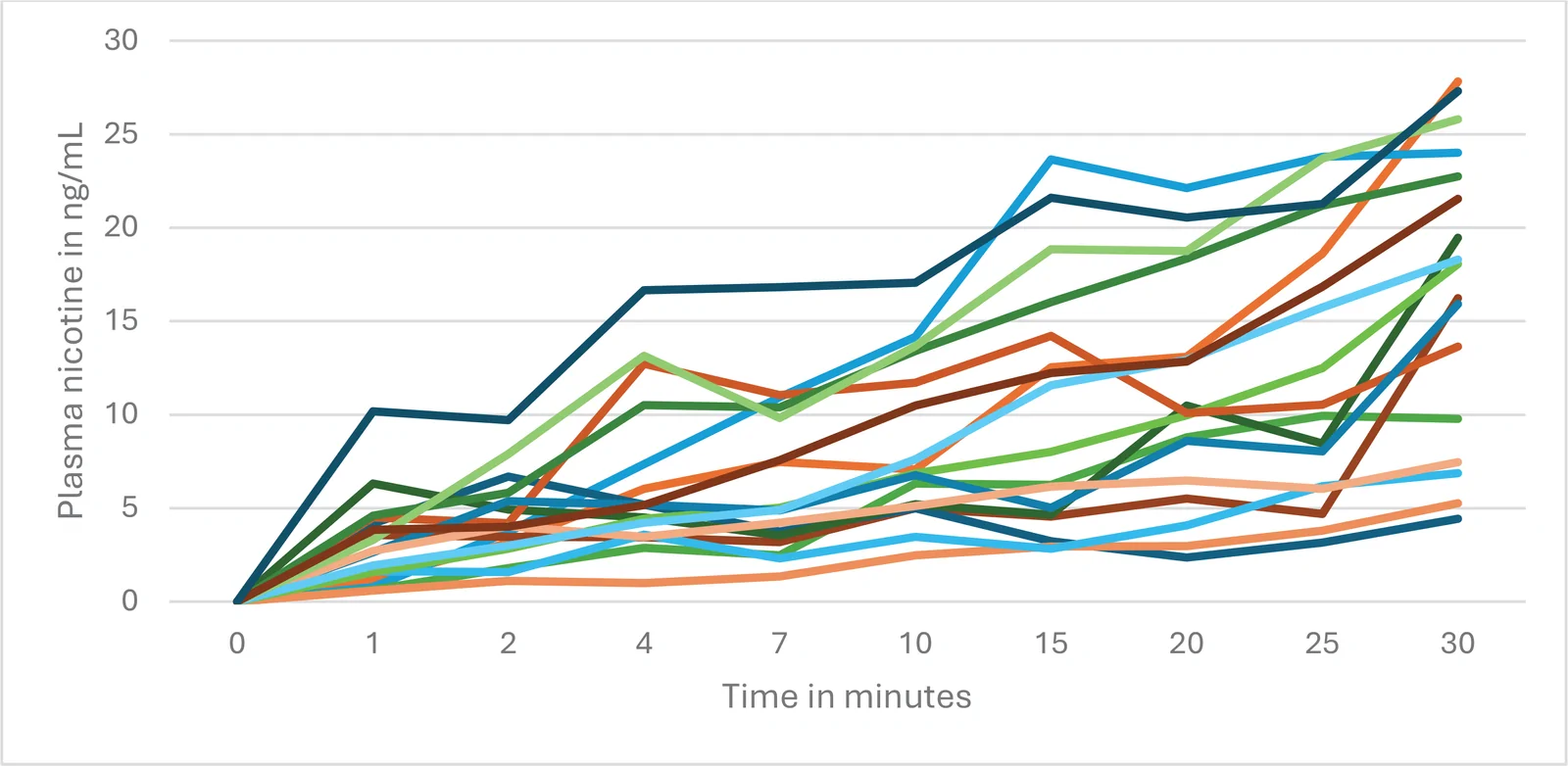
Nicotine delivery and concentration of individual participants measured over a 30-minute ad libitum consumption period in a single-center, one-arm prospective pre-post interventional study conducted at the Department of Psychiatry and Psychotherapy, LMU University Hospital, 2024 (N=17)

When comparing the nicotine delivery of e-cigarettes with that of tobacco cigarettes^15^, early-phase nicotine uptake from e-cigarettes is broadly comparable to that of tobacco cigarettes but may be modestly lower. Nicotine concentration consistently increases until the 30-minute mark with a final mean of 16.74 ng/mL.

### Puffing behavior

Puffing behavior was compared to raw data, of cigarette and 5-minute e-cigarette consumption, in an adjacent earlier study^15^. A Student’s t-test for independent samples revealed a significant difference between conventional tobacco cigarette and ad libitum e-cigarette consumption on all puffing parameters measured. At first it was observed that the number of puffs and interval between puffs differed (p=0.001). However, it is noteworthy that the differences in total volume and mean number of puffs remained relatively modest when comparing consumption duration of 5 versus 30 minutes. Specifically, the mean number of puffs (e-cigarette: 29.59; tobacco cigarette: 15.53) only approximately doubled, despite the elapsed time being six times longer. Mean flow per puff differed significantly between e-cigarette and tobacco cigarette (p<0.001), as did the duration per puff (p=0.004). Furthermore, mean and total puffing volume were significantly larger for e-cigarette than for tobacco cigarette (p<0.001).

In addition, smoking topography of ad libitum e-cigarette consumption was also compared to 5-minute e-cigarette consumption using Student’s t-test for independent samples. The puffing behavior differed significantly in the number of puffs (p=0.004) and interval between puffs (p=0.001). Similarly to the comparison with conventional cigarette consumption, the ratio of increase in number of puffs per time was relatively low. Specifically, the mean number of puffs (e-cigarette: 29.59; 5-min e-cigarette: 18.00) increased approximately 1.6-fold and the mean total inhaled volume (e-cigarette: 2323.07 mL; 5-min e-cigarette: 1318.63 mL) increased approximately 1.8-fold, despite the elapsed time being six-fold longer. While the mean puffing volume showed no significant difference between products, total puffing volume was significantly larger for e-cigarette use (p=0.005). Further, duration of puffs was significantly longer (p=0.020) than tobacco cigarette and a Mann-Whitney U test confirmed this finding (p=0.029). All parameters are to be found in the Supplementary file.

### Negative subjective effects

No major side effects were recorded during or after ad libitum e-cigarette consumption; all being described as minor. While there was no side effect which caused particular concern, the most frequent one was found to be throat irritation (four participants). A summary of negative subjective effects can be found in the Supplementary file.

## DISCUSSION

The present study characterizes a tobacco-naïve e-cigarette user population and compares the findings with data from a previous study, which assessed occasional smokers consuming a tobacco cigarette and a disposable e-cigarette, respectively^15^. Especially noteworthy, the nicotine dependence of tobacco-naïve e-cigarette consumers was queried and to the best of our knowledge this presents one of the first contributions in the literature finding clinical nicotine dependence within this population.

While both PSECDI and FTND indicated moderate to low nicotine dependence, this varied greatly between participants and especially the participants with higher test scores raise further concern. Furthermore, it is important to mention that participants frequently had difficulties recalling the extent of consumption, indicating lower bound estimates of actual dependence levels.

Another key finding was that both the questionnaires and the single item reveal a reduction in craving over the span of product use; however, the motivation to continue consumption remains. Craving decreased after consumption and indeed shows a negative association with nicotine in blood over time and hence an effective nicotine delivery.

The smoking topography observed in our ad libitum puffing regime free study showed a steady, less bolus type nicotine titration through a frequent an ongoing use. While e-cigarette users took more puffs in total than tobacco smokers, the ratio of puff frequency to session length is of interest, showing an elongated consumption pattern.

We observed a gradually increasing blood nicotine concentration throughout the 30-minute ad libitum consumption period. This pattern reflects ad libitum use, where individuals could consume e-cigarettes unrestricted, likely leading to steady, less frequent use, than a rapid peak.

This nicotine uptake pattern differs from that reported by Falarowski et al.^15^ on e-cigarettes and tobacco cigarettes, who demonstrated a quicker nicotine uptake rate for tobacco cigarettes. Peak nicotine concentrations were reached within the first few minutes of smoking. This rapid peak can be linked to the high nicotine concentration in the smoke and its immediate absorption in the lungs. Our findings suggest that while nicotine uptake from disposable e-cigarettes (ad libitum) occurs more gradually than from tobacco cigarettes, the overall nicotine concentrations can still reach similar levels over time^15^. The slower delivery may be less intense than the quick peak seen with cigarette smoking but as shown, can still lead to nicotine dependence with prolonged use. The ad libitum study design helps to better understand real-world e-cigarette usage patterns. Users may not always smoke continuously. Instead, they take intermittent puffs throughout the day. Despite the longer time frame, participants stated motivation to continue consumption past the 30-minute mark yet at that stage had reached comparably high nicotine delivery in blood as conventional cigarette smokers. Overall, due to the differing delivery profile, the results demonstrate the need for further research characterizing the consumption patterns of e-cigarette users. While ad libitum consumption can be considered more realistic, some e-cigarette users do describe product use in sessions, which could equate closer to the 5-minute time frame in our prior study^15^. More studies that provide robust evidence are needed, as oftentimes e-cigarette use is less heavily regulated as tobacco cigarette use, enabling a steadier, less bolus type nicotine titration through more frequent and ongoing use^27^. Further, the larger puffing volume may proof to be relevant when considering pulmonary risks as documented in related studies. Secondly, the comparison of ad libitum e-cigarette use to 5-minute e-cigarette use adds to this further. While seemingly trivial, the differing number and interval of puffs can be seen as evidence against sessions and points towards a steady and longer usage pattern. The smaller ratio in difference between number of puffs and total volume on the other hand could point towards stocking up behavior when consumption is restricted, i.e. before school, between work, or in restaurants. This idea is supported by the shorter duration of puffs, when restricted to 5 minutes, which could indicate a more rushed consumption. Therefore, we are arguing that 30-minute ad libitum consumption may be more representative of true consumption pattern in everyday life, yet we point out the need for even longer ad libitum studies to better conclude on nicotine titration^28^. These ideas are further supported by queried craving for nicotine. The reduced but continuous craving could be the underlying factor of the steady nicotine titration discussed. Craving is strongly related with both the intensity and frequency of consumption^29^. Consequently, a state of persistent, moderate craving after use seems concerning, as we suggest it may maintain current usage patterns to avoid withdrawal but also reinforces the psychological drive for following consumption. Lastly, when put into the context with queried addiction, these findings suggest a qualitatively different kind of addiction driven by a usage pattern that differs fundamentally from that of conventional tobacco cigarettes. More studies that provide robust data to support our findings are needed as well as a detailed characterization of a potentially qualitatively new kind of addiction along with adequately tailored cessation strategies. For example, most current standardized tobacco cessation programs identify situations and places associated with smoking^30^. E-cigarettes, however, are used in a wide variety of situations, potentially reducing the effectiveness of interventions that rely on identifying and avoiding specific triggers^31,32^.

### Limitations

The assessment of e-cigarette use, nicotine dependence, and adverse events relied on self-reported questionnaires, which are subject to recall bias, reporting bias, and social desirability bias. However, these data cannot be measured directly and are therefore commonly assessed using self-reported instruments. Furthermore, studies with larger sample sizes are needed to confirm our findings and we report a number of exclusions due to technical reasons and practical deviations during testing (like delays in measurements, subject drop out and baseline nicotine <10 ng/mL). Since acute effects were examined, we did not monitor participants after the last study visit. Consequently, no information on how their usage behavior changed after the study can be provided, which could add insight. Furthermore, the realistic context of the study slightly affected reliability as there was no discrimination of flavors. The single-center single-arm study design without an integrated control group represents a further limitation, as potential confounding factors could not be accounted for, causal interferences cannot be drawn and the generalizability is restricted. Lastly, though the comparison to data collected in the previous study by Falarowski et al.^15^ was handled with caution, this was not optimal due to the differing time frames.

## CONCLUSIONS

The role of e-cigarettes as an introductory product is becoming increasingly worrisome. With this in mind, it is essential to closely monitor usage trends and consider strengthening regulations, particularly within youth. The present study contributes to the still limited evidence on clinical nicotine dependence in tobacco-naïve individuals, thereby extending the ongoing debate on the risks associated with e-cigarettes. It demonstrates that dependence can develop independently of conventional cigarette consumption. Further research is needed to deepen our understanding of e-cigarette use trajectories and associated health risks among tobacco-naïve users. This research should move beyond comparative designs that treat e-cigarettes merely in relation to conventional smoking, but instead examine them as an independent issue domain.

## Supplementary Material



## Data Availability

The data supporting this research are available from the authors on reasonable request.
