# Supplementary material for: Nicotine dependence and smoking topography in tobacco-naïve users of disposable e-cigarettes: A prospective single-center, single-arm interventional study
Source: Tob Induc Dis. 2026 Nov 6;24:10.18332/tid/236207. doi: 10.18332/tid/236207 (PMC13741281; doi:10.18332/tid/236207)
Supplement: Supplementary file 1 [file TID-24-208-s1.pdf]

Tobacco Induced Diseases

Supplementary file

© 2026 Strohmeyer E. et al.

DOI:
10.18332/tid/236207

The content has been provided by the author(s) and has not been reviewed, verified, or endorsed by European Publishing. It may not have undergone peer review. The views, opinions, and recommendations expressed are solely those of the author(s) and do not necessarily reflect the position of European Publishing. European Publishing accepts no responsibility or liability for any consequences arising from the use of, or reliance on, this content.

Supplement

Hypotheses

1) The smoking topography of dependent e-cigarette users, consuming ad libitum for 30 minutes, differs from that of intermittent tobacco smokers and 5 minute e-cigarette consumption within:

- Puffing behaviour
- Number and volume of puffs
- Interval between puffs

2) The nicotine level after five minutes, corresponds to the values of conventional cigarette smokers.

3) Participants exhibit clinical addiction, measured by the Penn State Electronic Cigarette Dependence Index (PSECD) and the Fagerström Test of Nicotine Dependence (FTND).

4) Acute craving is significantly reduced after the consumption of e-cigarettes. Multiple self-ratings on a scale from zero to seven are used to operationalize this. In addition, the “Questionnaire on Smoking Urges” (QSU-U) is asked before and after consumption.

5) The side effect profile shows no major variations between baseline and end of consumption.

Inclusion criteria

a) Aged between 18 and 29 years
b) Minimum 12 hours nicotine abstinence prior to testing
c) Baseline nicotine plasma concentration < 10ng/mL
d) Informed consent
e) Use of disposables for minimum 3 months. Current consumption minimum 6 days/week.
f) Self-declaration as regular e-cigarette user.

Exclusion criteria

a) Aged below 18 or above 29 years.

b) Non-e-cigarette user or

c) Lifetime smoker (≥ 100 cigarettes and/or tobacco-containing joints in their lifetime)

d) Tobacco dependence according to ICD-10 / FTND > 1 or PSECD > 3

1) Daily cigarette consumption
2) Frequent consumption of other nicotine products

e) Existing pregnancy

1) For contraceptive methods with a Pearl Index > 0.9, a negative pregnancy test must be available before the start of testing.

f) Psychiatric illnesses

1) Suicidality
2) Acute psychiatric illnesses according to ICD-10
3) Drug, medication, or alcohol abuse at the time of the study

g) Previous internal medical conditions

1) Acute malignant cancer
2) Malignant cancer within the last 5 years
3) Severe previous conditions/risk factors of the cardiovascular system
   i. Manifest arterial hypertension
   ii. Severe pre-existing cardiac conditions (history of heart attack, heart failure)
   iii. Pacemaker
   iv. Diabetes mellitus
4) Respiratory diseases (e.g., asthma, chronic obstructive pulmonary disease (COPD))
5) Severe active infectious diseases (hepatitis, human immunodeficiency virus (HIV))

h) carbon monoxide (CO) > 5 ppm in exhaled air

i) Other circumstances that, deemed by the investigator, argue against participation in the present study

Participants

A total number of 29 individuals were screened, 12 of them were excluded: 1 met an exclusion criterion, 3 dropped out by mutual agreement, 6 had to be excluded due to technical problems, 2 due to too high baseline. To secure a tobacco abstinence of at least 12 hours, the participants' CO in the exhaled air was measured.

Table S1

Demographic characteristics of all participants of a prospective single-center, one-arm pre-post interventional study conducted in 2024 at the Department of Psychiatry and Psychotherapy LMU

Parameter	Total (n = 17) (mean ± SD)
Age (years)	21,82 ± 2,61
Female sex (%)	9 (53%)
Height (cm)	172,76 ± 8,66
Weight (kg)	75,59 ± 25,62
BMI (kg/m^2)	25,1 ± 7,5
Puffs per day	216,38 ± 186,10

Figure S1

Smoking Puff Analyzer – Mobile (SPA-M) device.

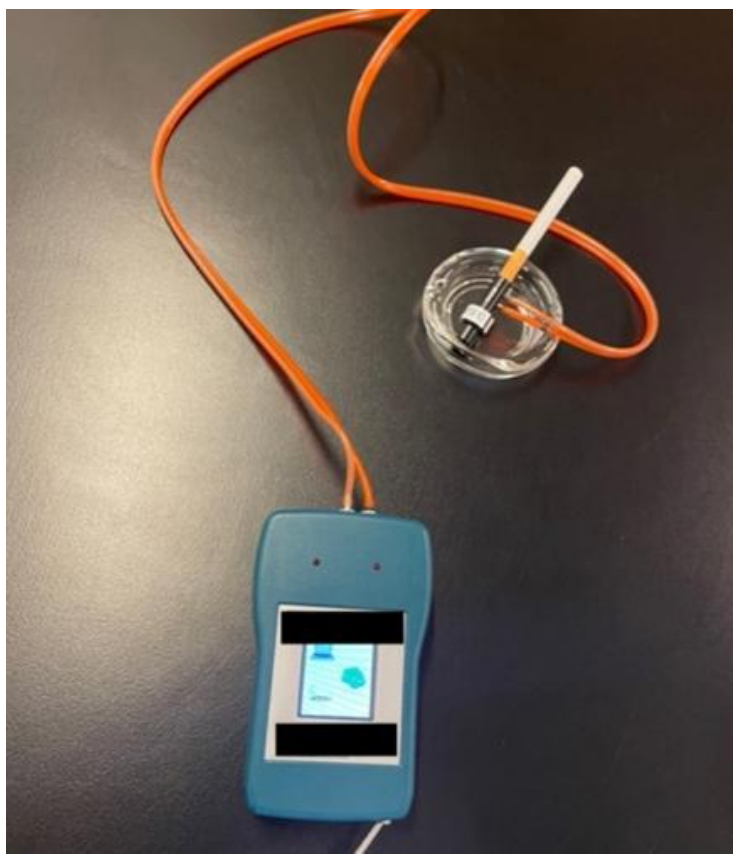

Figure S2

E-cigarette flavor selected by the participants.

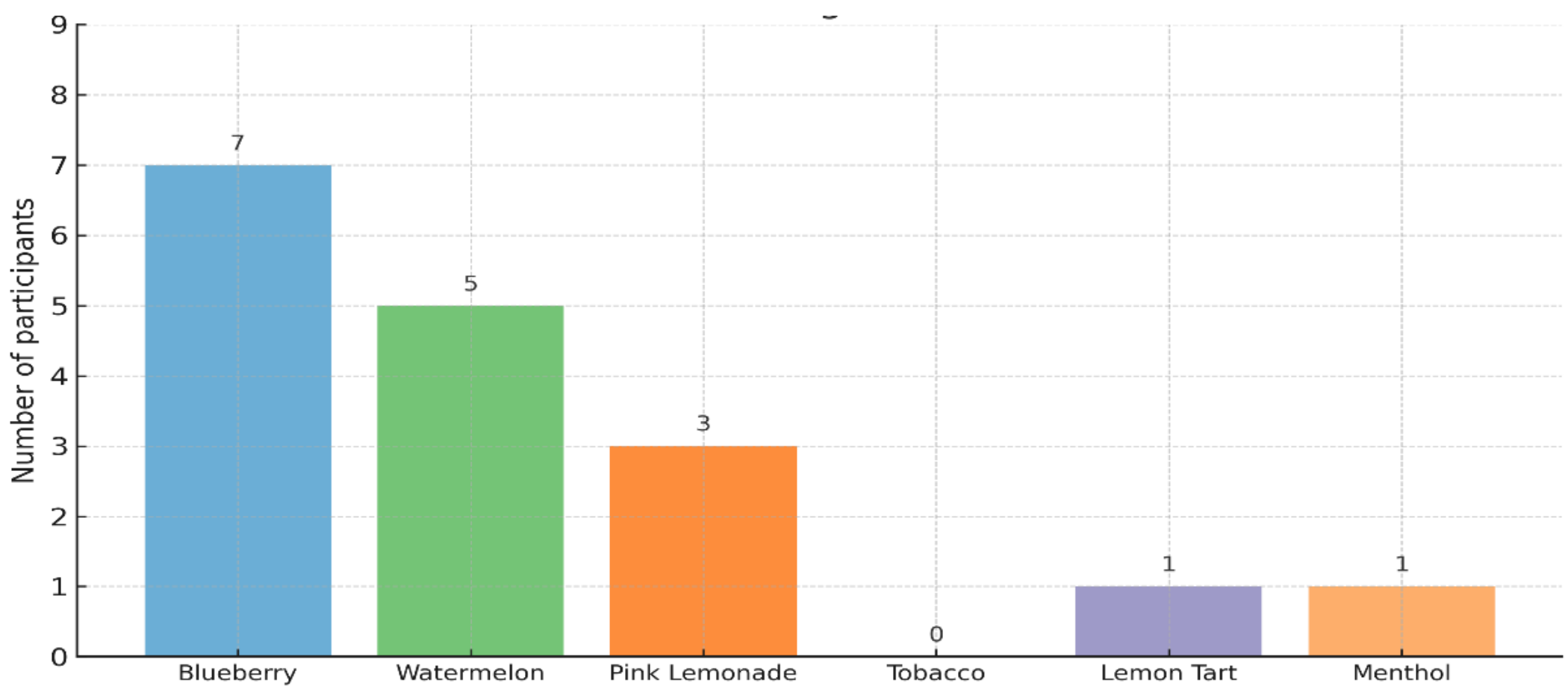

Table S2

Puffing topography comparison to data extracted from a recent adjacent study by Falarowski et al. [15].

	Group	n	Mean	Std. Deviation
Volume (total)	E-cigarette	17	2323.1	1190.4
	Tobacco cigarette	17	492.4	173.3
Volume (mean)	E-cigarette	17	89.3	48.9
	Tobacco cigarette	17	33.7	14.2
Duration of puffs (mean)	E-cigarette	17	2.4	.8
	Tobacco cigarette	17	1.7	.6
Flow per puff (mean)	E-cigarette	17	36.4	11.3
	Tobacco cigarette	17	22.6	8.8
Peak flow per puff (mean)	E-cigarette	17	51.9	15.9
	Tobacco cigarette	17	37.4	14.9
Interval between puffs (mean)	E-cigarette	17	69.4	54.6
	Tobacco cigarette	17	21.5	5.7
Number of puffs	E-cigarette	17	29.6	14.6
	Tobacco cigarette	17	15.5	3.6

	Group	n	Mean	Std. Deviation
Volume (total)	E-cigarette (30 min)	17	2323.1	1190.4
	Tobacco-flavored e-cig. (5 min)	18	1318.6	723.1
Volume (mean)	E-cigarette (30 min)	17	89.3	48.9
	Tobacco-flavored e-cig. (5 min)	18	78.9	45.1
Duration of puffs (mean)	E-cigarette (30 min)	17	2.4	0.8
	Tobacco-flavored e-cig. (5 min)	18	1.8	0.7
Flow per puff (mean)	E-cigarette (30 min)	17	36.4	11.3
	Tobacco-flavored e-cig. (5 min)	18	43.0	16.3
Peak flow per puff (mean)	E-cigarette (30 min)	17	51.9	15.9
	Tobacco-flavored e-cig. (5 min)	18	62.3	22.9
Interval between puffs (mean)	E-cigarette (30 min)	17	69.4	54.6
	Tobacco-flavored e-cig. (5 min)	18	16.3	5.7
Number of puffs	E-cigarette (30 min)	17	29.6	14.6
	Tobacco-flavored e-cig. (5 min)	18	18.0	6.2

Note. Smoking topography of e-cigarette users in this study compared to data from an adjacent study with 1) Tobacco smokers and 2) Tobacco flavored e-cigarette users, who were restricted to five-minute consumption. Duration measured in seconds, volume in milliliters.

Figure S3

Side effects recorded.

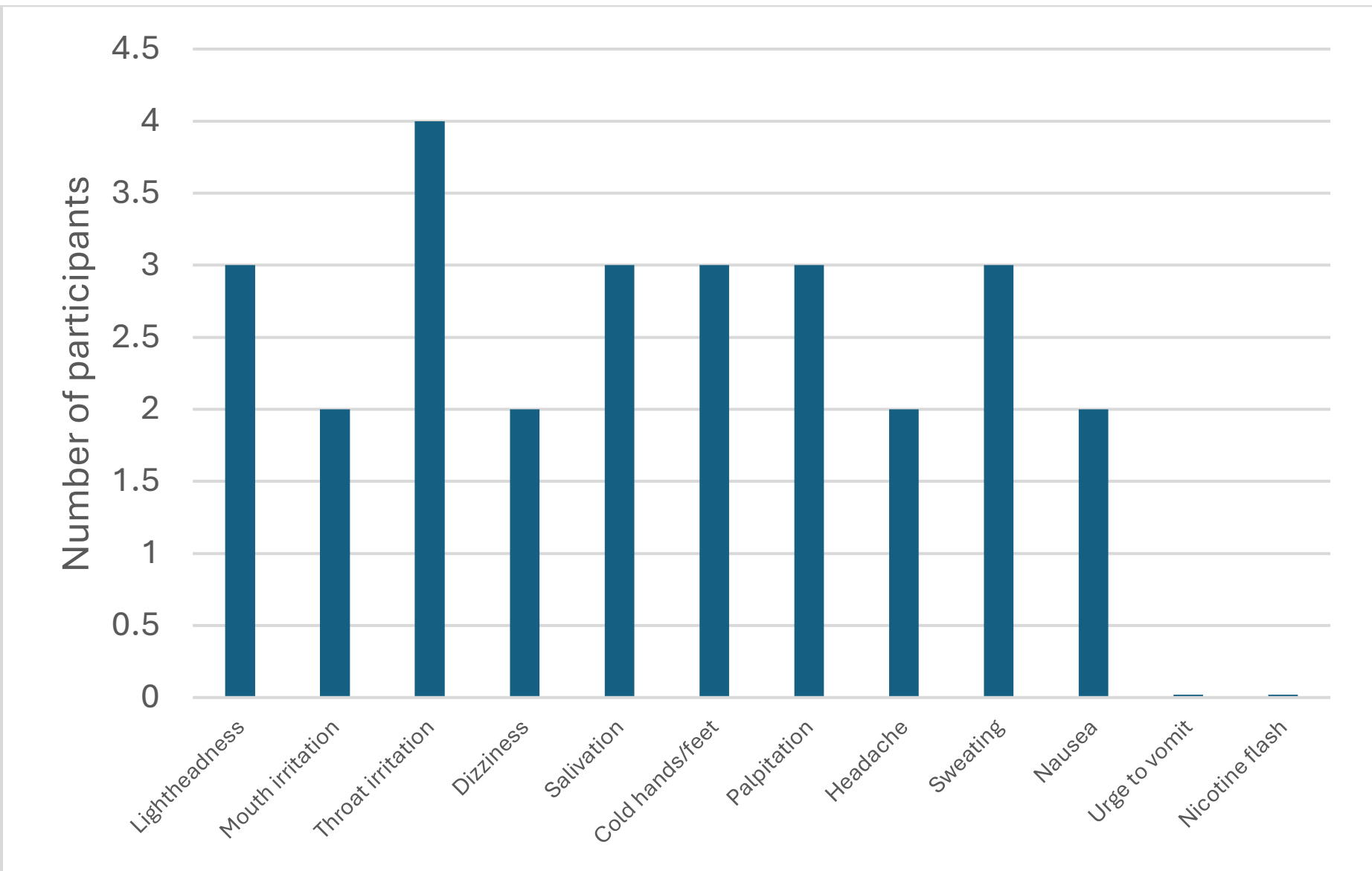

Preliminary examination form including FTND and PSECDI

Voruntersuchungs-Bogen

1. Allgemeines

1.1 Ihr Geschlecht: ☐männlich ☐weiblich ☐divers, bitte zusätzlich noch das biologische Geschlecht mit angeben.
(In einigen Geräten muss in den Einstellungen zwischen männlich und weiblich gewählt werden.)

1.2 Ihr Alter: ______ Jahre

1.3 Größe: ________cm

1.4 Gewicht: ________kg

1.5. Haben Sie seit mindesten 12 Stunden keine E-Zigarette gedampft, sind nikotinabstinent?
☐ja ☐nein

2. Dampferanamnese

2.1 Dampfen Sie täglich? Bzw. mindestens 6x pro Woche?
☐ja ☐nein

2.2 Würden Sie sich selbst als „regelmäßigen Dampfer“ bezeichnen?
☐ja ☐nein

2.3. Nutzen Sie seit mindestens 3 Monaten, an mindestens 6 Tagen die Woche Disposables?
☐ja ☐nein

2.4. Haben Sie in den letzten 30 Tagen ein anderes inhalatives Produkt konsumiert? (Dual-Use)
☐ja ☐nein

2.5. Haben Sie im letzten halben Jahr eine Shisha-Session gehabt, oder liegt ihr Lifetime Shisha Konsum über 5 Sessions?
☐ja ☐nein

2.6. Haben Sie in den letzten 30 Tagen eine Tabakzigarette geraucht, oder liegt ihr Lifetime Konsum über 100 Zigaretten?
☐ja ☐nein

3. Lifetime-Konsum

3.1. Seit wann dampfen Sie regelmäßig?

____

3.2. Wie oft pro Tag oder Woche dampfen Sie?

____

3.3. Wie lange dampfen Sie im Schnitt pro Session?

____

3.4. Welches Produkt bzw. welche Marke konsumieren Sie bevorzugt?

____

3.5. Konsumieren Sie sonstige nikotinhaltigen Produkte? Falls Ja, in welcher Frequenz (Anzahl/Monat)?

3.5.1 ☐ ja ☐ nein

3.5.2 ____

____

____

3.6. Welche Nikotinmenge ist in Ihrer E-Zigarette enthalten?

____

3.7. Möchten Sie ihr Rauchverhalten ändern?

☐ja ☐nein

3.8. Haben oder hatten Sie seit Sie vapen Symptome, die Sie auf die E-Zigaretten zurückführen:

☐ja ☐nein

3.8.1 Wenn ja, welche:

____

Wie lange habe diese angehalten:

3.8.2. Während des Konsums/Vapens: ☐

3.8.3. über die Dauer des Konsums/Vapens hinaus, für: ☐

3.8.4. ☐ für einen Tag ☐ mehrere Tage ☐ über eine Woche ☐ mehrere Wochen

4. <u>The E-cigarette Fagerström Test of Nicotine Dependence</u>

4.1 Wie schnell nach dem Aufwachen dampfen Sie zum ersten Mal?

- Innerhalb von 5 Minuten (3)
- 6-30 Minuten (2)
- 31-60 Minuten (1)
- Nach 60 Minuten (0)

4.2 Finden Sie es schwierig, auf das Dampfen zu verzichten, wenn es verboten ist?

- Ja (1)
- Nein (0)

4.3 Wann fällt es Ihnen besonders schwer auf das Dampfen zu verzichten?

- Das erste Mal Dampfen am Morgen (1)

- Jedes andere Mal (0)

4.4 Wie viele Pods (18mg/ml Nikotin I 0,7ml I 12,6mg Nikotin) dampfen Sie am Tag?

- 0-0,5 (0)
- 0,5-1 (1)
- 1-1,5 (2)
- 1,5 und mehr (3)

4.5 Dampfen Sie stärker in den ersten Stunden nach dem Aufstehen als während des übrigen Tages?

- Ja (1)
- Nein (0)

4.6 Dampfen Sie auch, wenn Sie so krank sind, dass Sie im Bett liegen müssen?

- Ja (1)
- Nein (0)

Gesamtpunktzahl: [__|__]

FTND: 0-2 geringe, 3-4 mittlere, 5-6 starke, 7-10 sehr starke körperliche Abhängigkeit

5. PSECDI

5.1 Wie oft am Tag verwenden Sie üblicherweise Ihre E-Zigarette? (Nehmen Sie an, dass eine Verwendung ca. 15 Züge beinhaltet bzw. ca. 10 min. dauert)

a) 0-4x /Tag (0)
b) 5-9x (1)
c) 10-14x (2)
d) 15-19x (3)
e) 20-29x (4)
f) ≥30x (5)

5.2 Wie schnell nach dem Aufwachen verwenden Sie Ihre E-Zigarette üblicherweise zum ersten Mal (an Tagen, an denen Sie die E-Zigarette frei verwenden können)?

a) 0-5 min (5)
b) 6-15 min (4)
c) 16-30 min (3)
d) 31-60 min (2)
e) 61-120 min (1)
f) ≥121 min (0)

5.3 Wachen Sie manchmal nachts auf, um Ihre E-Zigarette zu verwenden?

a) Ja (1)
b) Nein (0)

5.4 Wenn ja, in wie vielen Nächten pro Woche wachen Sie typischerweise auf, um Ihre E-Zigarette zu verwenden?

a) 0-1 Nächte	(0)
b) 2-3 Nächte	(1)
c) ≥4 Nächte	(2)

5.5 Verwenden Sie inzwischen eine E-Zigarette, weil es sehr schwer ist, mit E-Zigaretten aufzuhören?

a) Ja	(1)
b) Nein	(0)

5.6 Hatten Sie jemals das starke Verlangen, eine E-Zigarette zu verwenden?

a) Ja	(1)
b) Nein	(0)

5.7 Wie stark ist in der letzten Woche Ihr Drang gewesen, eine E-Zigarette zu verwenden?

a) Kein Drang/wenig	(0)
b) Moderat/stark	(1)
c) Sehr stark/extrem stark	(2)

5.8 Finden Sie es schwierig, an Orten, an denen Rauchen verboten ist, auf eine E-Zigarette zu verzichten?

a) Ja	(1)
b) Nein	(0)

5.9 Wenn Sie für einige Zeit keine E-Zigarette verwendet haben oder als Sie versucht haben damit aufzuhören...
Haben Sie sich reizbarer gefühlt, weil Sie keine E-Zigarette verwenden konnten?

a) Ja	(1)
b) Nein	(0)

5.10 Haben Sie sich nervös, unruhig oder ängstlich gefühlt, weil Sie keine E-Zigarette verwenden konnten?

a) Ja	(1)
b) Nein	(0)

5.11 Gesamtpunktzahl: [__|__]

(PSECDI: 0-3 nicht abhängig,4-8 geringe, 9-12 mittelgradige, ≥13 hohe Abhängigkeit)

6. Medizinische Vorgeschichte

6.1 Leiden Sie an sonstigen Erkrankungen / Allergien? Wenn ja an welchen?

____

6.2 Nehmen Sie regelmäßig (täglich) Medikamente ein? (freiwillige Angabe)
☐ja ☐nein

6.2.1 Wenn ja, gegen welche Beschwerden (+ Präparatname)?
☐ wegen Blutdruck-, Herzproblemen
Präparat: _
☐ wegen Schmerzen
Präparat: _
☐ wegen der Schilddrüse
Präparat: _
☐ wegen Problemen mit der Lunge oder den Bronchien
Präparat: _
☐ wegen Problemen mit den inneren Organen (Magen, Darm, Leber, etc.)
Präparat: _
☐ wegen Diabetes (Zucker) oder anderen Stoffwechselerkrankungen
Präparat: _
☐ wegen psychischen Problemen
Präparat: _
☐ sonstige Gründe: ____

Präparat: _

7. Geschmackssorte

Welche Geschmackssorte ELF BAR 600 wollen Sie bei Visit 2 dampfen? Bitte wählen Sie eine Sorte, die Ihrem gewöhnlichen Konsum sehr nah kommt.

- Blueberry raspberry – BERRIES
- Watermelon – FRUIT OTHER
- Pink Lemonade – OTHER BEVERAGES
- Tabak – TOBACCO
- Lemon Tart – DESSERT OTHER
- Spearmint – MENTHOL/MINT
